# The Digital Marshmallow Test (DMT) Diagnostic and Monitoring Mobile Health App for Impulsive Behavior: Development and Validation Study

**DOI:** 10.2196/25018

**Published:** 2021-01-22

**Authors:** Michael Sobolev, Rachel Vitale, Hongyi Wen, James Kizer, Robert Leeman, J P Pollak, Amit Baumel, Nehal P Vadhan, Deborah Estrin, Frederick Muench

**Affiliations:** 1 Cornell Tech Cornell University New York City, NY United States; 2 Feinstein Institute for Medical Research Northwell Health Great Neck, NY United States; 3 The Partnership to End Addiction New York, NY United States; 4 College of Health and Human Performance Department of Health Education and Behavior University of Florida Gainsville, FL United States; 5 University of Haifa Haifa Israel

**Keywords:** impulse control, impulsivity, self-regulation, self-control, mobile health, mHealth, ecological momentary assessment, active task, ResearchKit

## Abstract

**Background:**

The classic Marshmallow Test, where children were offered a choice between one small but immediate reward (eg, one marshmallow) or a larger reward (eg, two marshmallows) if they waited for a period of time, instigated a wealth of research on the relationships among impulsive responding, self-regulation, and clinical and life outcomes. Impulsivity is a hallmark feature of self-regulation failures that lead to poor health decisions and outcomes, making understanding and treating impulsivity one of the most important constructs to tackle in building a culture of health. Despite a large literature base, impulsivity measurement remains difficult due to the multidimensional nature of the construct and limited methods of assessment in daily life. Mobile devices and the rise of mobile health (mHealth) have changed our ability to assess and intervene with individuals remotely, providing an avenue for ambulatory diagnostic testing and interventions. Longitudinal studies with mobile devices can further help to understand impulsive behaviors and variation in state impulsivity in daily life.

**Objective:**

The aim of this study was to develop and validate an impulsivity mHealth diagnostics and monitoring app called Digital Marshmallow Test (DMT) using both the Apple and Android platforms for widespread dissemination to researchers, clinicians, and the general public.

**Methods:**

The DMT app was developed using Apple’s ResearchKit (iOS) and Android’s ResearchStack open source frameworks for developing health research study apps. The DMT app consists of three main modules: self-report, ecological momentary assessment, and active behavioral and cognitive tasks. We conducted a study with a 21-day assessment period (N=116 participants) to validate the novel measures of the DMT app.

**Results:**

We used a semantic differential scale to develop self-report trait and momentary state measures of impulsivity as part of the DMT app. We identified three state factors (inefficient, thrill seeking, and intentional) that correlated highly with established measures of impulsivity. We further leveraged momentary semantic differential questions to examine intraindividual variability, the effect of daily life, and the contextual effect of mood on state impulsivity and daily impulsive behaviors. Our results indicated validation of the self-report sematic differential and related results, and of the mobile behavioral tasks, including the Balloon Analogue Risk Task and Go-No-Go task, with relatively low validity of the mobile Delay Discounting task. We discuss the design implications of these results to mHealth research.

**Conclusions:**

This study demonstrates the potential for assessing different facets of trait and state impulsivity during everyday life and in clinical settings using the DMT mobile app. The DMT app can be further used to enhance our understanding of the individual facets that underlie impulsive behaviors, as well as providing a promising avenue for digital interventions.

**Trial Registration:**

ClinicalTrials.gov NCT03006653; https://www.clinicaltrials.gov/ct2/show/NCT03006653

## Introduction

### Background

The classic Marshmallow Test performed by Mischel and colleagues [1] determined that the inability to inhibit short-term responding in childhood was predictive of lower educational attainment, lower stress resilience, and higher drug use and BMI in adulthood. In this test, children were offered a choice between one small but immediate reward (eg, one marshmallow) or a larger reward (eg, two marshmallows) if they waited for a period of time. Despite future research suggesting that multiple factors such as socioeconomic status mediated the relationship between delayed gratification and life outcomes [2], the study instigated a wealth of research on the relationships among impulsive responding, self-regulation, and clinical and life outcomes [3-11].

Impulsivity is a multidimensional construct characterized primarily by the inability to inhibit responding for short-term rewards despite long-term negative consequences or loss of potential gains [12-14]. Impulsivity is a common transdiagnostic feature of many disorders in the Diagnostic and Statistical Manual [15]. A plethora of psychological and medical studies have demonstrated the relationship of impulsivity traits to a variety of physical and mental health outcomes [14,16]. Across studies and subtypes, highly impulsive individuals were found to be significantly more likely to suffer from obesity, type II diabetes, substance use disorder, attention deficit/hyperactivity disorder (ADHD), gambling problems, bipolar disorder, borderline personality disorder, and suicidal behaviors, among others [14,16,17]. Levels of impulsivity not only predict the onset of numerous conditions but also the likelihood of successful intervention outcomes [18-20].

### Measurement of Impulsive Behavior

Measurement of impulsivity has long been considered challenging in psychological and medical research due to the multidimensional nature and heterogeneous manifestations of the construct [13,14]. Impulsive behavior includes a number of related but distinct types of traits such as positive and negative urgency, lack of planning or premeditation, lack of perseverance, inattention, present and future discounting, response inhibition, and sensation seeking [13]. Evidence suggests that each of the subtypes of impulsivity manifests itself in different ways on self-report and neurobiological and cognitive measurements, and that different types of measurements have strengths and weaknesses in identifying underlying components of impulsive behaviors [21-23].

Consequently, relations between self-report and performance-based assessment are consistently of low magnitude, but are independently associated with cognitions and behaviors [21,24]. For example, a meta-analysis of the relationship between impulsivity and BMI found that performance-based behavioral measures of impulsivity yielded significantly larger effect sizes than questionnaires, and that different domains of impulsivity were independently associated with BMI [25]. Because these measures are not highly correlated but do predict different facets of impulsivity and clinical outcomes, assessment paradigms should include a wide range of assessments with the ability to personalize to the specific clinical context. This assessment methodology will increase diagnostic accuracy by predicting specific underlying facets to advance the science rather than focusing on a single construct of impulsivity [26].

A distinction between impulsivity as a personality type or trait exhibited over time and across contexts versus a temporary state influenced by substances and other stimuli also warrants examination [10]. In general, trait-based personality models of impulsive behavior reveal robust relationships with life outcomes [27] and symptomatology [16]. At the same time, trait-based studies can be confounded by other factors, including environment, mood, cognition, and social setting [2,28,29], and are heavily influenced by current state and context. Consequently, it is important to measure both trait and state impulsivity via self-report and behavioral measures over time to better understand the relationship to clinical outcomes in real-world settings [29]. The majority of trait and behavioral measures of impulsivity were not designed or validated as state measures or for use as part of a frequent monitoring assessment paradigm; however, several initial studies have revealed that impulsive behaviors can be reliably measured in real-world settings using ecological momentary assessment (EMA) and experience sampling [21,29-33].

### Mobile Health

Mobile health (mHealth) technology has demonstrated the ability of smartphone apps and sensors to collect data pertaining to individual activity, behavior, symptoms, cognition, and context [34-37]. mHealth research platforms and frameworks, including Apple’s ResearchKit (iOS) [38] and Android’s ResearchStack [39], provide the opportunity to develop novel and scalable mHealth studies utilizing a variety of patient-reported and generated data [40,41]. mHealth studies demonstrated the potential of collecting personalized and frequent multimodal data in the lived experience of individuals to enhance the assessment, monitoring, and diagnostics of medical conditions, and to reveal symptom clusters [42,43].

mHealth technology can further advance the science of impulsivity by increasing the accuracy with which impulsivity as a whole can predict negative outcomes such as onset or exacerbation of psychiatric or medical conditions and treatment failure. mHealth apps can greatly facilitate intensive longitudinal studies [44,45] to understand within-subject differences in impulsive behaviors in everyday contexts. Multimodal methods for studying the underlying constructs of impulsivity separately combine behavioral and self-report measures, and include both trait- and state-based methods to enable a more comprehensive and frequent assessment of the facets of impulsivity. Each of these trait and state measures of impulsive behavior can be further personalized and adapted to individuals and to the context of the study. This personalized and modular approach is particularly useful for the study of impulsive behaviors as they are common in clinical trials of physical, medical, and psychological conditions.

To expand the measure of impulsivity, we developed mobile versions of validated laboratory assessments of impulsivity to be performed on a mobile phone along with daily and momentary self-report measures using Apple’s ResearchKit (iOS) [38] and Android’s ResearchStack [39] mHealth platforms. We combined these measures with traditional self-report and laboratory measures of impulsivity in a comprehensive study called the Digital Marshmallow Test (DMT).

### Objective

The primary aim of this study was to advance the science of impulsivity and the study of impulsive behavior by developing and refining a mobile diagnostic and monitoring app using trait- and state-based self-report and performance measurements of the underlying facets of impulsivity. To achieve this goal, we conducted a 21-day intensive longitudinal study measuring facets of impulsivity using the mobile DMT app.

## Methods

### DMT App

#### Overall App Design

**Figure 1:**
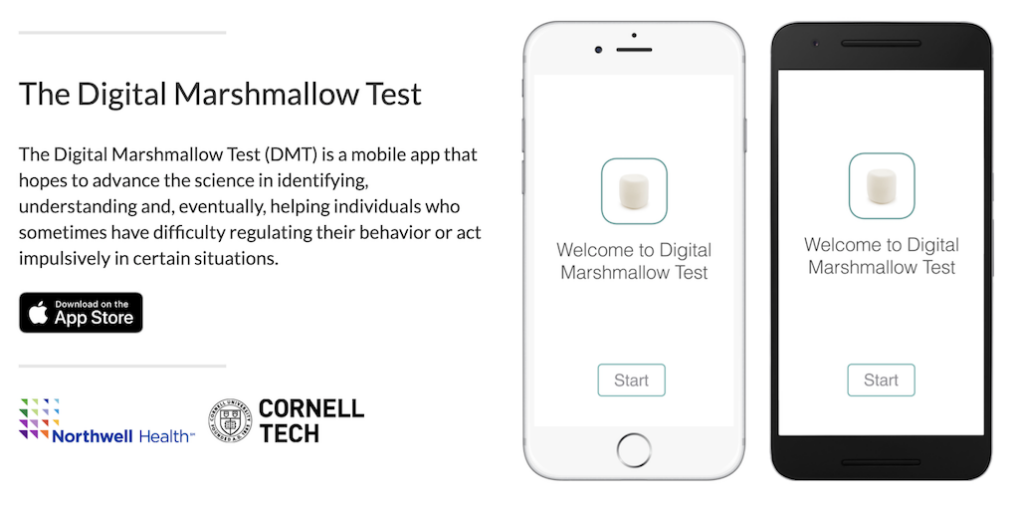
Digital Marshmallow Test (DMT) mobile apps for Apple (iOS) and Android.

**Figure 2:**
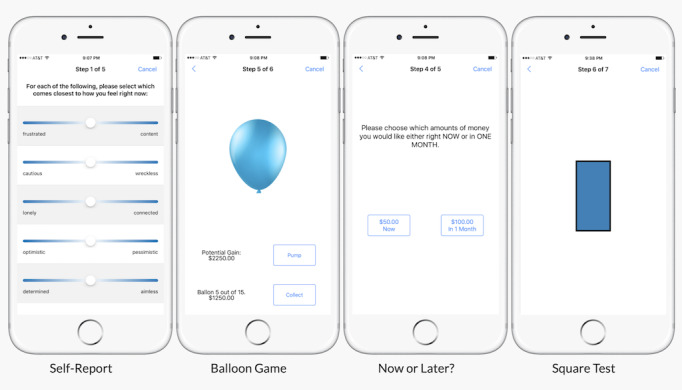
Active performance tasks and self-report in the Digital Marshmallow Test (DMT) app.

We developed a mobile monitoring app for remote assessment and monitoring of impulsive behavior called the DMT. The DMT app was developed based on Apple’s ResearchKit (iOS) and Android’s ResearchStack open source frameworks for developing health research study apps (Figure 1), which allow for researchers to easily develop intuitive and standardized data-collecting mobile apps. The DMT app consists of three main modules: baseline self-report, EMA of the current state, and active behavioral performance tasks (Figure 2).

#### Self-Report

Self-report data and patient-reported outcomes are ubiquitous in behavioral and medical research. Self-report measures of personality and traits are common in assessments of impulsivity in clinical trials and practice [29]. We collected a variety of clinically relevant self-report measures and outcomes using semantic differentials [46], general trait measures of impulsivity [13,47], and daily measures of impulsivity [29] as described below.

#### EMA

EMA methods involve repeated sampling of subjects’ current behaviors and experiences [48]. EMA measures are commonly used in clinical trials and mHealth research. For impulsivity, EMA methodology can be used to understand intraindividual variability and the situational factors of impulsive behavior [29,49]. Our DMT app includes a variety of EMA questions based on the semantic differential scale [46,50] that are prompted in the morning and the evening every day.

**Figure 3:**
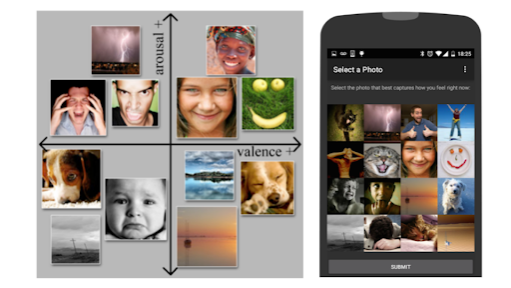
Photographic Affect Meter (PAM) for ecological momentary assessment.

We also implemented the Photographic Affect Meter (PAM; Figure 3) to measure emotional state and affect. The PAM is designed for assessing momentary response in which users choose an image that best represents their emotion at a given time [51]. We used the positive and negative affect scores from the PAM that had been validated to correspond to the Positive and Negative Affect Schedule (PANAS) [52].

#### Active Performance Tasks

Active performance tasks are some of the more innovative parts of Apple’s ResearchKit (iOS) [38] and Android’s ResearchStack [39] open source frameworks. These tasks invite users to perform activities under partially controlled conditions while phone sensors are used to collect data. ResearchKit [38] includes several predefined documented tasks developed by Apple and the research community [53], which fall into categories such as motor activities, fitness, cognition, and speech. ResearchStack supports a wide variety of community-contributed apps, although at the time of writing there is no centralized listing or repository of these tasks. In the cognition category, one relevant example is the adaptation of the classic Stroop Color and Word Test that is widely used in clinical practice and psychological research [54].

As part of the DMT app and study, we adapted three relevant behavioral and cognitive performance tasks to mobile devices. Specifically, we adapted three laboratory behavioral measures related to impulse control: mobile Balloon Analogue Risk Task (mBART), mobile Go-No-Go (mGNG), and mobile Delay Discounting (mDD). These tasks were modified visually to conform to mobile phone specifications, and were adapted to be used daily to measure behavioral manifestations of impulse control and behavior. For example, the mBART presented users with 15 balloons in each trial and took about 2 minutes to complete (Figure 3). Additional details on the development of active tasks can be found in the DMT project folder at the OSF [55].

#### ResearchKit and ResearchStack

We developed DMT using an extension of Apple's ResearchKit (iOS) and Android’s ResearchStack open source frameworks for developing research study apps, which allow researchers to easily develop intuitive and standardized data-collecting mobile apps. These platforms are designed to meet the requirements of most scientific research, including capturing participant consent, extensible input tasks, and the security and privacy needs necessary for Institutional Review Board approval. The extension was built on top of ResearchKit and ResearchStack, and extends the available surveys to include adaptable visual assessments and custom performance tasks such as the BART, and supports integration of the Ohmage-Omh [56,57] backend out of the box. Other server integrations can easily be created, such as the Sage Bionetworks Bridge Platform [40].

**Figure 4:**
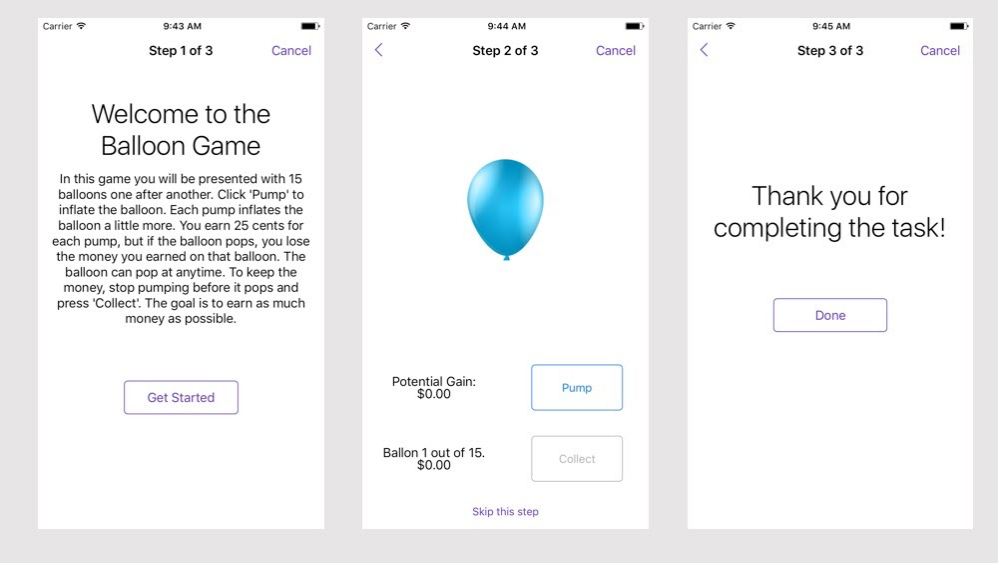
Example of an active task: mobile Balloon Analogue Risk Task (mBART).

The structure of an app is defined by a JavaScript Object Notation (JSON) file, which specifies the survey or active task steps to be instantiated by the app. The JSON file is converted into an array of Step objects, which the app uses to create a task using the Task Builder that is then presented to the research participant. The results of the task are handled by the Results Processor, which includes modules for storing the results locally and emailing them to the researcher, sending them to the Ohmage-Omh study manager, or sending them to a custom server. For example, to create the mBART for DMT (Figure 4), a researcher would need to create a JSON file [55]. The mBART consists of three steps: (1) an instruction step introducing the study, (2) the mBART active step, and (3) a final instruction step thanking the participant.

#### Testing

Ten beta users tested app functionality between August and November 2016. Both iOS and Android platforms were tested, feedback was provided to the developers, and a second version of the app was released in December 2016. The final version used for the study was released in January 2017.

### DMT Study

#### Participants

Participants were recruited from the Genotype and Phenotype Registry [58], a genetics bank initiated and managed by the Feinstein Institute for Medical Research at Northwell Health [59]. Participants completed a brief anonymous online screening assessment, which indicated whether or not they were eligible to participate in the study. Eligible participants then completed a phone call with a researcher, which involved a general overview of informed consent and scheduling a one-time in-person appointment at the Northwell Health lab. All study data were sent to a HIPAA (Health Insurance Portability and Accountability Act)-compliant database server provided by Sage Bionetworks. This study was approved by the Feinstein Institute of Medical Research within the Northwell Health Institutional Review Board [55].

Eligibility criteria for this study consisted of being fluent in, and able to read, English at the eighth grade level, being between the ages of 18 and 75 years, and owning a smartphone. Individuals who reported serious mental or physical health concerns as evidenced by current treatment or threshold symptoms over the past year were not included in outreach emails. Mental health concerns included any form of psychosis or psychotic disorder, bipolar disorder, and major depression. Participants received US $50 for their baseline interview, US $25 for completing at least 80% of their morning and evening assessments, and US $25 for the day-21 survey.

The total sample size was 116, with 63.8% (n=74) women and a mean age of 44.7 (SD 13.92) years. Overall, 70.7% (82/116) of the participants identified as White, 10.3% (12/116) as Black/African American, 7.8% (9/116) as Hispanic/Latinx, and 11.2% (13/116) as other. The sample was mixed in terms of education, with 36.2% (42/116) having less than a college degree, 27.6% (32/116) having a college degree, and 36.2% (42/116) having a graduate degree. Among the 116 participants, 85 (73.3%) were employed and 58 (50.0%) were married. The average BMI was 28.1 (SD 6.86) kg/m^2^. Attrition was relatively low compared to other mHealth studies [60]. Of the 116 participants recruited, 104 (89.7%) completed the mobile baseline assessment, 100 (86.2%) completed at least one morning and one evening assessment, and 93 (80.2%) completed the day-21 assessment.

#### Procedure

During the in-person appointment at the Northwell Health lab, participants were able to address any concerns pertaining to the study, including smartphone usage and privacy. The appointment was then divided into three parts. First, subjects completed the standard self-report and behavioral measures on a computer (see below). In the second part of the appointment, participants were instructed on how to download the DMT app onto their smartphones and were shown a 5-minute training video on how to use the app, as well as what was expected of their participation throughout the 21-day study. After participants watched the training video and had the opportunity to ask any questions, they completed the baseline assessment on the DMT app. Additional details on the procedure and materials of the study can be found in the DMT project folder at the OSF [55].

#### Laboratory Assessments

##### Trait Self-Report

We used two of the most common generalized impulsivity questionnaires: the Barratt Impulsiveness Scale (BIS) and the Urgency, Premeditation (lack of), Perseverance (lack of), Sensation Seeking, Positive Urgency Impulsive Behavior Scale (UPPS).

The BIS is the most widely cited instrument for the assessment of impulsiveness, and has been used to advance understanding of this construct and its relationship to other clinical phenomena [47,61]. We used a shorter version called BIS-15 [62]. The BIS-15 scale measures three aspects of impulsivity: attention (inability to focus attention or concentrate), motor (to act without thinking), and nonplanning (lack of future orientation or forethought).

The UPPS Impulsive Behavior Scale [13] assesses impulsivity on the subscales of urgency (acting rashly under conditions of negative affect), lack of premeditation (difficulty in thinking and reflecting on consequences of an act), lack of perseverance (inability to remain focused on a task), and sensation seeking (tendency and openness to try and enjoy exciting or dangerous activities). These subscales have a heterogeneous relationship with psychopathology [16]

##### Behavioral and Cognitive Active Performance Tasks

We used validated standard versions of three behavioral measures commonly used to measure impulse control and related constructs: BART, GNG, and DD. These measures are computerized and were performed at the Northwell Health lab.

BART is a measure of risk-taking that requires individuals to balance the potential for reward and loss via repeated opportunities to earn virtual money by pumping a balloon [63]. The standard BART has been found to predict risk-taking behavior, substance misuse, gambling, and unhealthy eating [63,64]. We used Inquisit software [65] with a script to measure impulsivity and risk aversion based on Lejuez et al [63], which has been validated in previous studies. Participants were presented with 30 balloons, one at a time. For each balloon they had the opportunity to repeatedly pump up the balloon to increase their potential hypothetical earnings, or to stop pumping and collect their accumulated earnings. However, if the balloon pops, the participant loses all of their potential winnings for the current balloon. The average number of pumps for unexploded balloons is the main dependent variable in this paradigm, with higher numbers indicating increased risk-taking. The standard laboratory BART task takes approximately 7 minutes to complete. We recorded the average number of pumps across all trials as a measure of risk-taking proclivity [63].

GNG is a measure of behavioral inhibition and cognitive control. Studies have found that individuals with ADHD display worse inhibitory control compared to controls [66]. GNG performance also differs between healthy controls and substance users or individuals with disordered eating [67,68]. We used Inquisit software [65] with an implemented procedure based on Fillmore et al [69]. Participants were asked to press the spacebar when they see a green rectangle (=go) but to refrain from pressing the spacebar when they see a blue rectangle (=no-go). The blue and green rectangles can be vertical or horizontal. The vertical rectangle has a high probability of being green (a “go” trial) and the horizontal rectangle has a high probability of being blue (a “no-go” trial). Participants receive information about the orientation of the rectangle (=cue) shortly before the color of the rectangle is revealed. Activation and inhibitory tendencies develop cue-dependence consistently [70]. The task included 250 cues and took approximately 10 minutes to complete. We recorded inhibition commission and omission errors jointly, and reaction time for responses to the targets across all trials.

DD is a measure of the ability to delay immediate smaller virtual rewards for delayed larger rewards. DD is a transdiagnostic process in psychiatric disorders [71,72]. We used the laboratory-based Inquisit software with an implemented procedure based on Richards et al [73]. Participants were asked to choose between either a standard amount of hypothetical money (US $10) with different time or probability delays or a variable amount with no delay until an indifference point for each delay is found, or until 30 trials have been run for each delay. This script establishes (1) the hypothetical payoffs at which participants start to discount higher monetary rewards in favor of shorter wait periods (delay discounting), and (2) the fictional payoffs at which participants start to discount high monetary rewards of unsure events in favor of lower monetary rewards of sure wins (probability discounting). The task takes approximately 15 minutes to complete. We recorded the cumulative probability of choosing the smaller reward across all 5 trials for each task administration [74,75].

#### Mobile Assessments (DMT App)

##### Schedule

We conducted a mobile study with a 21-day assessment period using the DMT app. The schedule of mobile assessments using the DMT app is summarized in Table 1.

**Table 1:** Digital Marshmallow Test (DMT) app assessment schedule during the 21-day study.

Assessment item		Baseline	Morning	Evening	Day 21
Self-report: feel in general (semantic differential items 1-20)		X			X
**Ecological momentary assessment**					
	Feel right now (semantic differential items 4, 5, 7, 8, 11, 12, 15, 17, 19, 20)		X	X	
	Photographic Affect Meter	X	X	X	
**Active Task**					
	mBART^a^	X	X^b^	X^b^	X
	mGNG^c^	X	X^b^	X^b^	X
	mDD^d^	X	X^b^	X^b^	X

^a^mBART: mobile Balloon Analogue Risk Task.

^b^Randomly display one out of mBART, mGNG, mDD.

^c^mGNG: mobile Go-No-Go.

^d^mDD: mobile Delay Discounting.

##### DMT Self-Report and EMA

The DMT app primary measures of impulsivity were assessed via self-report semantic differentials [46,50]. We selected 20 items from the semantic differential scale and used a selection of items at different time points during the study as described in Table 1. All of the items were measured both at baseline and at the end of the study. Participants were asked to either report semantic differentials based on trait (feeling in general) or current state (feel right now).

The DMT app also prompted the PAM (Figure 3) to measure emotional state and mood. The PAM is designed for momentary response where users choose an image that best represents their emotion at a given time [51]. The PAM was prompted at baseline, every morning and evening, and at the end of study (Table 1).

##### DMT Active Performance Tasks

The DMT app version of the BART (mBART) was similar to the laboratory version except that it was shorter (15 trials; about 2 minutes long). Participants were instructed to earn as much money as possible during the 15 trials (Figure 4). For participants, the task was named the “Balloon Game” (Figure 2).

The DMT app version of the cued GNG (mGNG) included 75 trials, each of which had the following sequence: fixation cross (250 milliseconds); blank screen (250 milliseconds), vertical or horizontal cue (white rectangle) for 1 of 6 stimulus onset asynchronies (100, 200, 300, 400, 500, 750 milliseconds); go or no-go target (green or blue rectangle, respectively) until the participant responds or for 500 milliseconds; and an intertrial interval (250 milliseconds). Participants were instructed to respond by pressing the screen as fast as possible to green, but not to blue, targets. Cues signal a target at 70% probability (horizontal: go, vertical: no-go). For participants, the task was named the “Square Test” (Figure 2).

In the DMT app version of the DD task (mDD), participants were given 5 choices between a smaller hypothetical monetary or time-based reward that varied from trial to trial based on the previous response and a larger fixed reward that remained the same throughout all trials. For participants, the task was named “Now or Later?” (Figure 2).

## Results

### Approach and Descriptive Statistics

In this study, we validated self-report, EMA, and behavioral measures of impulsive behavior on mobile devices. Overall, we validated our mobile assessments against previously validated clinical measures of impulsivity such as the BIS-15 and UPPS. We also examined the psychometric properties of our novel measures. Descriptive statistics and analyses are available in the DMT project folder at the OSF [55].

### Semantic Differentials

We performed a principal component analysis with varimax rotation of the 20-item semantic differential scale that was measured at baseline (Table 1). Our exploratory analysis yielded a solution with 6 factors of traits we called inefficient, negative, calm, unhealthy, thrill-seeking, and intentional. Combined, these components explained 74% of the variance in the scale. Full results of the principal components analysis are shown in Table 2.

**Table 2:** Factor loadings of principal component analysis of the semantic differential scale at baseline.

Semantic differentials	Inefficient	Negative	Calm	Unhealthy	Thrill	Intentional
1. Efficient-Inefficient	*0.849^a^*	0.146	–0.055	0.129	–0.054	–0.009
2. Organized-Unorganized	*0.821*	–0.026	–0.044	–0.073	0.172	–0.080
3. Productive-Unproductive	*0.765*	0.289	0.017	0.318	–0.031	0.062
4. Focused-Distracted	*0.721*	0.209	–0.337	0.135	–0.052	–0.229
5. Determined-Aimless	*0.580*	0.534	–0.063	0.176	–0.091	0.069
6. Clear headed-Confused	*0.580*	0.210	–0.348	0.128	–0.149	–0.375
7. Bored-Engaged	–*0.539*	–0.184	0.392	–0.229	0.015	0.272
8. Optimistic-Pessimistic	0.133	*0.867*	–0.122	0.016	–0.072	0.075
9. Positive-Negative	0.249	*0.837*	–0.156	–0.056	0.040	0.090
10. Sad-Happy	–0.052	–*0.682*	0.527	–0.245	0.063	0.087
11. Lonely-Connected	–0.015	–*0.654*	0.311	–0.280	–0.145	0.135
12. Proud-Ashamed	0.460	*0.646*	–0.063	0.113	–0.001	–0.035
13. Calm-Anxious	0.227	0.094	–*0.836*	–0.106	–0.037	0.013
14. Stressed-Relaxed	–0.071	–0.239	*0.835*	–0.216	–0.141	–0.071
15. Frustrated-Content	–0.073	–0.444	*0.597*	–0.125	–0.132	0.138
16. Healthy-Unhealthy	0.139	0.118	–0.028	*0.882*	0.002	–0.047
17. Energetic-Tired	0.373	0.122	–0.235	*0.708*	–0.139	0.163
18. Conservative-Progressive	0.024	–0.006	–0.166	–0.147	*0.795*	0.239
19. Cautious-Thrill seeking	–0.039	0.029	0.045	0.065	*0.784*	–0.341
20. Impulsive-Intentional	–0.178	0.070	0.011	0.077	–0.059	*0.908*
Explained variance (%) (α)	20 (.88)	18 (.87)	13 (.81)	9 (.71)	7 (.46)	7 (N/A^b^)

^a^Values in italics indicate factors corresponding to the component.

^b^N/A: not applicable.

### Correlations with BIS-15 and UPPS

We examined correlations between validated measures (BIS-15 and UPPS) and our 6 factors. The inefficient and thrill-seeking factors were highly correlated with various trait measures of impulsivity and impulsive behavior. In contrast, the negative, calm, and unhealthy factors showed only minimal or nonsignificant correlations with trait measures of impulsivity and impulsive behavior. The *impulsive-intentional* factor, which consists of only one item, was significantly correlated with 7 out of 9 trait-based measures. Full results of correlations are shown in Table 3.

**Table 3:** Correlations between semantic differential factors and the Barratt Impulsiveness Scale (BIS-15)/Urgency, Premeditation (lack of), Perseverance (lack of), Sensation Seeking, Positive Urgency Impulsive Behavior Scale (UPPS).

Variable		Inefficient		Negative		Calm		Unhealthy		Thrill		Intentional	
**Motor**													
	*r*		0.320		0.008		–0.216		–0.068		0.106		–0.534
	*P* value		.001		.94		.03		.50		.29		<.001
**Nonplanning**													
	*r*		0.352		–0.012		0.097		0.059		–0.098		–0.234
	*P* value		<.001		.90		.33		.55		.33		.02
**Attention**													
	*r*		0.423		0.209		–0.307		0.067		–0.055		–0.150
	*P* value		<.001		.03		.002		.50		.58		.13
**BIS-15**													
	*r*		0.516		0.097		–0.188		0.033		–0.031		–0.416
	*P* value		<.001		.33		.06		.74		.76		<.001
**Urgency**													
	*r*		0.376		0.115		–0.146		0.105		–0.023		–0.419
	*P* value		<.001		.25		.14		.29		.82		<.001
**Premeditation**													
	*r*		0.278		–0.035		–0.050		0.058		0.324		–0.304
	*P* value		.004		.72		.61		.56		.001		.002
**Perseverance**													
	*r*		0.575		0.150		–0.206		0.094		0.019		–0.062
	*P* value		<.001		.13		.04		.34		.85		.53
**Sensation seeking**													
	*r*		–0.082		–0.136		0.068		0.026		0.542		–0.361
	*P* value		.41		.17		.49		.79		<.001		<.001
**UPPS**													
	*r*		0.346		0.008		–0.094		0.101		0.359		–0.477
	*P* value		<.001		.94		.34		.31		<.001		<.001

### Intraindividual Variability

We further examined the intraindividual variability in self-reported semantic differentials between baseline and morning and evening measures. We compared how individuals’ “feeling in general” self-reports correlated with average daily reports of the same semantic differentials over 21 days.

As shown in Table 4, correlations between baseline and morning measures ranged from moderate (*r*=0.4) to high (*r*=0.7) with *lonely-connected*, *optimistic-pessimistic*, and *determined-aimless* having the highest correlations, and *focused-distracted*, *energetic-tired*, and *bored-engaged* having the lowest correlations. Correlations between baseline and evening measures ranged from low (*r*=0.2) to high (*r*=0.7) with *lonely-connected*, *cautious-thrill seeking*, and *impulsive-intentional* having the highest correlations, and *focused-distracted*, *energetic-tired*, and *bored-engaged* having the lowest correlations. Overall, the deviations and variability from baseline were similar across morning and evening momentary measures, with evening demonstrating lower calibration with baseline measures.

Correlations between morning and evening measures were very high (*r*=0.8-0.9). We found that individuals were more impulsive, distracted, aimless, tired, pessimistic, and thrill-seeking in the evening compared to the morning. Otherwise, we found that individuals reported similar levels of boredom, loneliness, ashamedness, and frustration in the mornings and evenings.

**Table 4:** Correlations and paired t test results between baseline, morning, and evening with semantic differentials.

Semantic differentials	Baseline vs morning			Baseline vs evening			Morning vs evening		
	*r*	*t* (df=97)	*P* value	*r*	*t* (df=98)	*P* value	*r*	*t* (df=97)	*P* value
Focused-Distracted	0.355	–2.313	.02	0.177	–3.864	<.001	0.820	–4.744	<.001
Determined-Aimless	0.548	–6.357	<.001	0.422	–8.308	<.001	0.836	–5.447	<.001
Bored-Engaged	0.391	0.582	.56	0.319	1.298	.20	0.808	1.741	.09
Optimistic-Pessimistic	0.574	–1.089	.28	0.481	–1.950	.05	0.916	–2.752	.007
Lonely-Connected	0.701	0.000	>.99	0.693	–0.111	.91	0.902	–0.702	.48
Proud-Ashamed	0.529	–3.526	.001	0.459	–3.007	.003	0.911	1.150	.25
Frustrated-Content	0.549	0.604	.55	0.514	0.081	.94	0.875	–1.201	.23
Energetic-Tired	0.492	–2.747	.007	0.273	–5.584	<.001	0.590	–5.254	<.001
Cautious-Thrill seeking	0.534	–0.350,	.73	0.539	–1.096	.28	0.911	–2.255	.03
Impulsive-Intentional	0.531	–0.279	.78	0.531	1.424	.16	0.872	4.545	<.001

### Effect of Emotional State and Affect

We examined four metrics from the PAM task (valence, arousal, positive, and negative) as they related to momentary semantic differentials in the morning and evening (Table 5). Across the four PAM metrics, positive affect generally correlated higher than others with various semantic differentials. Across the 10 semantic differentials examined, *energetic-tired* and *frustrated-content* showed the highest correlations with PAM metrics. However, both *impulsive-intentional* and *cautious-thrill seeking* semantic differentials did not generally correlate with any of the PAM metrics (Table 5).

**Table 5:** Correlations between semantic differential factors and Photographic Affect Meter measures.

Semantic differentials		Valence			Arousal			Positive			Negative	
		Morning	Evening	Morning		Evening	Morning		Evening	Morning		Evening
**Focused-Distracted**												
	*r*	–0.392	–0.406	–0.448		–0.333	–0.475		–0.461	0.244		0.283
	*P* value	<.001	<.001	<.001		.001	<.001		<.001	.02		.004
**Determined-Aimless**												
	*r*	–0.365	–0.452	–0.500		–0.371	–0.468		–0.513	0.197		0.314
	*P* value	<.001	<.001	<.001		<.001	<.001		<.001	.05		.001
**Bored-Engaged**												
	*r*	0.317	0.425	0.551		0.425	0.442		0.508	–0.128		–0.264
	*P* value	.001	<.001	<.001		<.001	<.001		<.001	.21		.008
**Optimistic-Pessimistic**												
	*r*	0.434	–0.422	–0.413		–0.236	–0.525		–0.442	0.331		0.337
	*P* value	<.001	<.001	<.001		.02	<.001		<.001	.001		.001
**Lonely-Connected**												
	*r*	0.434	0.445	0.483		0.361	0.522		0.504	–0.275		–0.311
	*P* value	<.001	<.001	<.001		<.001	<.001		<.001	.006		.002
**Proud-Ashamed**												
	*r*	–0.449	–0.585	–0.453		–0.347	–0.526		–0.620	0.302		0.460
	*P* value	<.001	<.001	<.001		<.001	<.001		<.001	.002		<.001
**Frustrated-Content**												
	*r*	0.578	0.607	0.390		0.361	0.618		0.644	–0.461		–0.477
	*P* value	<.001	<.001	<.001		<.001	<.001		<.001	<.001		<.001
**Energetic-Tired**												
	*r*	–0.479	–0.461	–0.706		–0.722	–0.630		–0.639	0.239		0.182
	*P* value	<.001	<.001	<.001		<.001	<.001		<.001	.02		.07
**Cautious-Thrill seeking**												
	*r*	–0.012	–0.065	0.079		0.104	0.014		–0.021	0.043		0.108
	*P* value	.90	.52	.44		.31	.89		.84	.68		.29
**Impulsive-Intentional**												
	*r*	0.190	0.322	0.008		0.078	0.167		0.303	–0.197		–0.299
	*P* value	.06	.001	.94		.44	.10		.002	.05		.003

### Active Performance Tasks

#### mBART

To validate the mBART, we assessed the correlation between behavior in the validated laboratory measure of BART and the exploratory mBART active task. The number of explosions in the lab (N=114; mean 6.55, SD 5.25) was highly correlated (*r*=0.658, *P*<.001) with the number of explosions in the mBART (N=102; mean 5.62, SD 2.65) at baseline. We also estimated the test-retest reliability of the number of explosions in the mBART and found high correlations between baseline and morning (*r*=0.663, *P*<.001), evening (*r*=.0673, *P*<.001), and day 21 (*r*=0.451, *P*<.001). Results for the number of pumps were almost identical to the results for the number of explosions, as these measures are highly correlated (*r*=0.643, *P*<.001). The number of explosions on mBART moderately correlated with the sensation-seeking trait (*r*=0.216, *P*=.03). Both the number of explosions (*r*=0.30, *P*=.002) and the number of pumps (*r*=0.268, *P*=.006) on the mBART correlated with the thrill-seeking factor from semantic differentials.

#### mGNG

To validate the mGNG, we tested the correlation between behavior in the validated laboratory measure of GNG and the exploratory mGNG active task. Response time (in milliseconds) in the lab (N=109; mean 353, SD 43) was highly correlated (*r*=0.467, *P*<.001) with response time in the mGNG (N=97; mean 430, SD 80) at baseline. We also estimated the test-retest reliability of response time in the mGNG and found high correlations between baseline and morning (*r*=0.88, *P*<.001), evening (*r*=0.862, *P*<.001), and day 21 (*r*=0.789, *P*<.001). Error rates between the lab and mobile version were not correlated due to the low overall error rate in the lab task (mean 0.00765, SD 0.014569) and the high overall rate of error in the mGNG (mean 0.39, SD 0.74). Notably, average error rates on the mGNG at baseline did not correlate with morning, evening, and day-21 error rates. The test-retest reliability changed during the study since morning correlated with evening (*r*=0.477, *P*<.001) and day 21 (*r*=0.454, *P*<.001), which also correlated with evening (*r*=0.461, *P*<.001). This is consistent with the participants’ reported frustration with mGNG during the study, which might have led to poorer performance. The response rate on the mGNG task negatively correlated with the sensation-seeking trait (*r*=–0.310, *P*=.002). The error rate on mGNG marginally negatively correlated with the organization factors from semantic differentials (*r*=–0.194, *P*=.06) and response time marginally negatively correlated with the cautious factor from semantic differentials (*r*=–0.196, *P*=.05).

#### mDD

We had trouble validating the mDD active task with the equivalent lab version, as we used a shortened exploratory version of the DD [74]. However, our results yielded moderate test-retest reliability and convergent validity. We examined the propensity of choosing the later reward with respect to both money and time. The propensity to choose the later reward (money) in 6 months correlated highly with the propensity of choosing the later reward (money) at 1 month (*r*=0.489, *P*=.002) and the later reward (time) in 1 year (*r*=0.396, *P*<.001). The propensity to choose the later reward (time) in 1 year highly correlated with the propensity to choose the later time reward in 6 months (*r*=0.523, *P*=.001). We also estimated the test-retest reliability of the propensity to choose later in the mDD and found high correlations. Propensity to choose the later reward (money) in 6 months at baseline correlated highly with the propensity of choosing the later reward (money) in 6 months at day 21 (*r*=0.414, *P*<.001). The propensity to choose the later reward (time) in 12 months at baseline correlated highly with the propensity of choosing the later reward (time) in 12 months at day 21 (*r*=0.394, *P*<.001). The propensity to choose the later reward (money) in 6 months at day 21 correlated highly with the propensity of choosing the later reward (time) in 12 months at day 21 (*r*=0.411, *P*<.001). There was no association between the mDD and any self-report measure.

## Discussion

### Principal Results

Overall, the present study demonstrated the potential for assessing different facets of trait and state impulsivity during everyday life using the DMT mobile app. Similar to previous research, the results suggest varying levels of concurrent and predictive validity between existing self-report measures and computer performance tasks, and mobile state and trait versions of these tasks measured over a 21-day period.

### Trait and State Self-Report Measures

We built on the semantic differential scale to develop self-report trait and state measures of impulsivity in the DMT app. Our exploratory principal component analysis of the baseline semantic differentials yielded six factors of trait impulsivity: inefficient, negative, calm, unhealthy, thrill-seeking, and intentional. We found that inefficient, intentional, and thrill-seeking factors were highly correlated with various facets of trait impulsivity, whereas the negative, calm, and unhealthy factors only slightly correlated with trait-based measures. Notably, the *impulsive-intentional* factor, which consists of only one item, significantly correlated with 7 out of 9 trait-based measures (BIS-15/UPPS) and can be potentially used as a parsimonious single-item measure of trait impulsivity.

To enhance understanding of state impulsivity and intraindividual variability, we examined the differences between general self-reports and momentary measures of semantic differentials in the morning and evening over the duration of the DMT study. Correlations between baseline and morning measures ranged from moderate (*r*=0.40) to high (*r*=0.70), with *lonely-connected*, *optimistic-pessimistic*, and *determined-aimless* showing the highest correlations, and *focused-distracted*, *energetic-tired*, and *bored-engaged* showing the lowest correlations. Correlations between baseline and evening measures ranged from low (*r*=0.20) to high (*r*=0.70), with *lonely-connected*, *cautious-thrill seeking*, and *impulsive-intentional* showing the highest correlations, and *focused-distracted*, *energetic-tired*, and *bored-engaged* showing the lowest correlations. Overall, the deviations and variability from baseline were similar across morning and evening momentary measures, with evening responses demonstrating lower calibration with baseline measures.

Our study design also allowed us to investigate these constructs in the context of daily life by comparing morning and evening momentary self-reports. Correlations between morning and evening measures were very high (*r*=0.80-0.90). We found that individuals were more impulsive, distracted, aimless, tired, pessimistic, and thrill-seeking in the evening compared to the morning. Otherwise, we found that individuals report similar levels of boredom, loneliness, ashamedness, and frustration in mornings and evenings. These results help highlight variations in the facets of impulsivity across the day. Measures that can be attributed to physical and mental depletion [76] had the most variability from morning to evening, whereas those that assess trait-based characteristics were more stable. It is important to recognize that we used a nonclinical sample. Previous studies (eg, Tomko et al [29]) reported that daily impulsivity may vary more in clinical samples than nonclinical samples, suggesting the need for further study in clinical populations.

The results also suggest that some momentary state assessments are highly related over time and day such as *focused-distracted* and *determined-aimless*, whereas others such as *lonely-connected* and *frustrated-content* revealed no significant relationships across all assessment periods. It is also noteworthy that some items, including *impulsive-intentional* and *thrill seeking-cautious,* were only correlated in the morning and evening versus from baseline to morning or evening, suggesting that although the means may vary from morning to evening, there is a relative intraday association and stability versus over time.

Finally, we examined the role of emotional state, including valence, arousal, positive, and negative, using the PAM [51]. We observed that positive affect generally correlated higher than valance, arousal, and negative affect with various momentary semantic differentials. Specifically, *energetic-tired* and *frustrated-content* showed the highest correlations with PAM metrics. However, both *impulsive-intentional* and *cautious-thrill seeking* semantic differentials did not generally correlate with any valence, arousal, positive, or negative measures of emotional state. These high correlations suggest that using self-report photos instead of or combined with text-based self-report items may enable expanding momentary state assessment to wider audiences regardless of language or education [51]. Further research is warranted to test the PAM in clinical samples across various cohorts.

### Active Performance Tasks

One of the primary goals of the DMT study was to validate the behavioral and cognitive active performance tasks in the DMT app. Previous research has highlighted the transdiagnostic potential of behavioral tasks [71,72] but has also identified challenges in test-retest reliability compared to self-reports [77]. In the DMT app, we modeled the design of the DMT active performance tasks (mBART, mGNG, and mDD) based on validated computerized versions of these tasks [63,69,74]. Despite the effort to match the mobile tasks to laboratory tasks, we found only moderate success in validation of these tasks. In this study, mBART demonstrated the highest validity, followed by mGNG and then mDD with the lowest validity.

Specifically, the mBART active task showed high correlations with the lab BART task, high test-retest reliability, and convergent validity with self-report measures. Risk taking in the mBART task correlated with self-reported sensation seeking, which corresponds to prior research with the lab-based BART [78]. Our results also correspond to those of MacLean and colleagues [79] who revealed that a different mobile version of the BART demonstrated good concurrent and predictive validity with the lab computer version. Unlike our results, which were mostly stable across administrations both regarding time of day and over time, there were some differences in BART indices over time in their sample of nondaily smokers. When the studies are combined, it appears that the BART can be translated to a mobile phone to reliably assess risk taking in real-world settings. Nevertheless, the weak correlation between self-report and behavioral measures of risk, as found in other studies [78,80], warrants future investigation of domain-specific or more general measures of risk [80] in the context of impulsive behavior.

GNG is a common behavioral measure of inhibition and cognitive control in clinical trials [66-69]. In these trials, two primary outcomes are usually used to measure cognitive control: error rate and response time. Error rate is particularly important in addiction and substance use studies. On mobile devices, and when performed in a natural setting and outside of the lab (ie, mGNG), the distribution of these metrics is expected to change dramatically. Our results suggest that reaction time is stable across time and contexts with correlations across baseline, morning, evening, and day 21 ranging from 0.79 to 0.86 with no significant mean differences. Error rates had less robust associations across time points, but overall means were relatively stable. This finding may be due to a floor effect in the computerized lab version of the GNG task, which is common in healthy samples and in clinical samples at baseline and without experimental manipulation (eg, alcohol administration) [81-83].

DD is used to measure the ability to delay immediate, smaller, shorter rewards for longer, time-lapsed, but larger rewards. In this study, we did not manage to obtain concurrent validity of the novel mDD and the lab DD task. Nevertheless, individual choices in the mDD during the DMT study showed moderate convergent validity via the correlation between DD money and time versions. Choices in the mDD also showed moderate test-retest reliability from baseline to day 21. The null findings might also be due to the hypothetical, as opposed to incentive-compatible, structure of the mDD task, which decreases validity [84,85], or use of the brief version of the task consisting of only 5 decision points.

When taken together, our results highlight that laboratory mobile assessments can be reliably collected in the field. Although there are some concerns over the relatively weak relationships with self-report impulsivity measures, except for the mBART, similar results have been found with previously validated computer versions of these tasks performed at baseline, suggesting more systemic problems in the objective measurement of impulsivity [23,26]. At the moment, these problems do not appear to be solved through the mobile versions of these tasks. We plan to refine and further validate the mBART, mGNG, and mDD tasks in future studies of the DMT app.

### Comparison With Other mHealth Apps and Related Studies

One common clinical use of mHealth apps is remote diagnosis [86]. A systematic review of direct-to-consumer apps identified lack of sufficient clinical evidence for many symptom checkers and diagnostic apps [87]. Our study, which combined validated assessments and novel measures, generated evidence to support the diagnostic capabilities of the DMT app. In the future, the DMT app can be used as a remote patient-facing mHealth app to diagnose and monitor impulsive behaviors.

Our goal was to develop and validate the DMT app for both researchers and clinicians. We used Apple’s ResearchKit [38] and Android’s ResearchStack [39] open source frameworks for developing health research study apps (Figure 1), which allow researchers to easily develop intuitive and standardized data-collecting mobile apps. The DMT app and measures we developed are cross-platform, open source, and standardized. Our mBART and mGNG tasks, for example, can be easily adapted by other researchers across a variety of psychological, behavioral, and clinical studies that use mobile devices.

Our study also suggests broader design implications for behavioral and cognitive active performance tasks in mHealth studies and apps. In these tasks, users perform activities under partially controlled conditions while phone sensors are used to collect data. User interfaces and user experience on mobile devices and apps are dramatically different from validated laboratory behavioral and cognitive tasks. Mobile performance tasks are often performed, as intended, in the lived experience of individuals with limited attention and ample distractions. Some tasks such as the mGNG and mDD in this study require more sustained concentration and information processing, while others such as the mBART are more engaging and gamified. The effect of user experience provides a challenge to validation studies, and requires more careful design of behavioral and cognitive active performance tasks in mHealth studies.

### Future of Impulsivity Assessments and Interventions

Despite the predictive power of laboratory and self-report measures on trait impulsivity, more research is needed with different samples to disentangle the relationship between impulsivity and health outcomes [2,28,29]. Our study revealed similar results to previous studies that impulsivity is not a unitary construct but is rather composed of qualitatively different constructs, which may or may not have some overlap [22]. Moreover, the complex relationship between impulsive behaviors and health outcomes within each individual might require an n-of-1 approach to prediction and control of impulsive behaviors [37,88,89]. New mHealth methods such as the DMT using multimodal assessment strategies that take trait and state impulsivity into account with contextual variables are needed to further our understanding of how to predict impulsive behavior. Future studies should account for contextual factors such as setting, mood, and intentionality to further disentangle the relationship between trait and state impulsivity, and the different dimensions measured by these tools. Contextual factors can also be used to design more precise behavior change and digital health interventions with mobile technology [88,90-93].

Our ultimate goal is to move from measurement of trait and state impulsivity toward implementation and evaluation of interventions for impulse control and behavior. Despite the overwhelming research on the impact of impulsivity on mental and physical health outcomes, it has been largely ignored as a target of intervention in its own right. Our mobile-based measures can be used to design personalized and adaptive interventions on the same mobile devices and app. Just-in-time adaptive interventions (JITAI) can be designed to provide the right type/amount of support, at the right time, by adapting to an individual’s changing internal and contextual state [90,94].

Research performed with daily self-report measures revealed that fluctuations in certain state impulsivity domains (eg, lack of planning, negative urgency) predict heavy drinking, highlighting the opportunity to trigger intervention based on day-to-day fluctuations [95]. Similarly, behavioral active tasks can detect deterioration in inhibitory control during the day [96]. To design JITAI, the combination of the single-item intentionality measurement with results from the mBART could potentially predict a vulnerable state of reduced intentionality and more risk taking on a particular morning compared to other days, or data trends that reveal slow changes in these variables over time. Consequently, with more research, DMT can potentially serve as a just-in-time intervention system for people who are prone to impulsivity and could be made available to people around the world.

Subsequently, understanding the user’s state using a game-like component can inform the design of new digital psychological interventions, since the same component could be used both for assessment and intervention seamlessly. For example, upon failure during the mBART, the user can potentially be directed to interact with a new balloon in a way that may help them reassess the number of pumps that may result in an explosion, in the same fashion that new health video games assess and adapt to the user state in an ongoing manner for the enhancement of therapeutic impact [97]. Similarly, research on interventions that manipulate discounting identified learning-based interventions as the most effective [98]. Using DMT to combine both assessment and intervention within one component opens up room for digital microinterventions that focus on very small and beneficial steps that people can take in their daily life [99], which may be far more acceptable than traditional long-term interventions. We plan to introduce and study different personalized and adaptive digital interventions [91,92,99,100] to reduce impulsive response in future studies of the DMT app.

### Limitations

The design and implementation of the current DMT study was not sufficient to fully validate the behavioral and cognitive active performance tasks we developed for the DMT app. Similar to other studies that have created mobile versions of impulsivity assessments, this is another step in the right direction despite limitations. In particular, we emphasize our limitation in validating the mDD against equivalent objective laboratory tasks due to challenges with both the lab task we selected and the mobile task we developed. We will continue in our effort to further refine and validate the mobile self-reports and active performance tasks against clinical symptom profiles, diagnoses, contextual factors, and behaviors to generate data on how these constructs are related to mental health and everyday life interactions.

This study was also substantially burdensome for participants due to the sheer number and frequency of required daily assessments. Our results will help to design a lean and personalized version of the DMT app for future studies as we attempt to replicate and further refine our measures. Finally, we plan to validate the DMT app in clinical samples in the context of obesity, addiction, and mental health.

### Conclusions

The DMT app can be used to enhance our understanding of impulsivity, impulsive behavior, and failure in self-regulation. Impulsivity measurement is a complex undertaking because of the multidimensionality of the construct as highlighted by the range of measures that assess multiple distinct components. Adding to this problem of construct validity are the various modes of assessment (eg, self-report versus behavioral active performance tasks) and the increased use of daily and momentary assessments. These challenges also present an opportunity to hone our assessment strategies.

Eventually, the goal is to use trait- and state-based self-report and behavioral measures to predict global and momentary clinical outcomes that can trigger personalized and adaptive digital interventions. These interventions can be targeted and tailored to reduce the various underlying triggers of impulsive responding and enhance self-regulation. Only through rigorous innovation and testing can we begin to build these timely interventions.

## Abbreviations

ADHD: attention deficit/hyperactivity disorder
BART: Balloon Analogue Risk Task
BIS: Barratt Impulsiveness Scale
DD: Delay Discounting
DMT: Digital Marshmallow Test
EMA: ecological momentary assessment
GNG: Go-No-Go
JITAI: just-in-time adaptive interventions
JSON: JavaScript Object Notation
mBART: mobile Balloon Analogue Risk Taker
mDD: mobile Delay Discounting
mGNG: mobile Go-No-Go
mHealth: mobile health
PAM: Photographic Affect Meter
UPPS: Urgency, Premeditation (lack of), Perseverance (lack of), Sensation Seeking, Positive Urgency Impulsive Behavior Scale
