# Correction: The Digital Marshmallow Test (DMT) Diagnostic and Monitoring Mobile Health App for Impulsive Behavior: Development and Validation Study

**DOI:** 10.2196/27439

**Published:** 2021-01-26

**Authors:** Michael Sobolev, Rachel Vitale, Hongyi Wen, James Kizer, Robert Leeman, J P Pollak, Amit Baumel, Nehal P Vadhan, Deborah Estrin, Frederick Muench

**Affiliations:** 1 Cornell Tech Cornell University New York City, NY United States; 2 Feinstein Institute for Medical Research Northwell Health Great Neck, NY United States; 3 The Partnership to End Addiction New York, NY United States; 4 College of Health and Human Performance Department of Health Education and Behavior University of Florida Gainsville, FL United States; 5 University of Haifa Haifa Israel

In “The Digital Marshmallow Test (DMT) Diagnostic and Monitoring Mobile Health App for Impulsive Behavior: Development and Validation Study” (JMIR Mhealth Uhealth 2021;9(1):e25018) the authors noted one error.

In the originally published article, in the subsection “Measurement of Impulsive Behavior,” a URL was inadvertently inserted following the sentence “At the same time, trait-based studies can be confounded by other factors, including environment, mood, cognition, and social setting [2,28,29], and are heavily influenced by current state and context.” This URL was unrelated to the article and has been removed from the corrected version.

The correction will appear in the online version of the paper on the JMIR Publications website on January 26, 2021, together with the publication of this correction notice. Because this was made after submission to PubMed, PubMed Central, and other full-text repositories, the corrected article has also been resubmitted to those repositories.

